# The role of multiparametric magnetic resonance imaging and magnetic resonance-guided biopsy in active surveillance for low-risk prostate cancer: A systematic review

**DOI:** 10.1016/j.amsu.2020.07.015

**Published:** 2020-07-17

**Authors:** Sultan Zaher Alshehri, Omar Safar Alshahrani, Nazal Ahmed Almsaoud, Muhammad Ahmad Al-Ghamdi, Abdulaziz Mohammed Alqahtani, Muath Mohammed Almurayyi, Ali Salem Autwdi, Saeed Ahmed Al-Ghamdi, Mohammed Mesadef Zogan, Abdulrahim Mohammed Alamri

**Affiliations:** a Department of Urology, Aseer Central Hospital, Abha, Saudi Arabia; b Department of Urology, Armed Forces Hospital Southern Region, Khamis Mushait, Saudi Arabia; c Department of Urology, King Fahad Central Hospital, Jazan, Saudi Arabia; d Department of Urology, King Fahad Hospital, Al Baha, Saudi Arabia

**Keywords:** MRI, MRGB, Active surveillance, Prostate cancer

## Abstract

The performance of multiparametric magnetic resonance imaging (mpMRI) and subsequent biopsy in monitoring prostate cancer in men on active surveillance (AS) have not been defined clearly. In this systematic review, we aimed to review current literature about the usage of MRI examination in men with low-risk prostate cancer during active surveillance. For that, we searched seven databases to include all studies reporting magnetic resonance imaging in the AS of low-risk prostate cancer. We finally included 11 studies with 1237 patients included. Our results showed an adequate sensitivity and specificity of both modalities to detect disease progression; including disease upgrading and upstaging. However, the performance in the prediction of unfavorable disease was inferior to the detection of upgrading and upstaging. In terms of MRGB, the previous literature agreed on the superiority of using a combination of different biopsy schemes to get a better progression section. Noteworthy, mp-MRI and MRGB had a good predictive value limited to the first year, with TRUSGB showing a superior role in detecting patients with a GS ≥ 7, after that. In conclusion, both of mpMRI and MRGB have shown an adequate performance on assessing disease progression in the AS of low-risk prostate cancer patients. They can be used for disease staging and grading for successful treatment planning.

## - HIGHLIGHTS

• In comparison to the literature, few papers discuss the benefit of MRI screening in low-risk prostate cancer groups.
• Biopsy is considered more invasive than MRI, thus reducing the burden of such methods on the patients.
• PSA values can be misinterpreted especially that it can rise in other diseases such as Benign Prostatic Hyperplasia.

## 1 Introduction

During the past decade, massive improvement has been implicated for more understanding of the epidemiology, diagnosis, and treatment of non-communicable diseases among different worldwide populations. Prostate cancer is a disease of men and considered to be a global health issue among the clinical society that interferes with the men's quality of life [1]. Prostate cancer is prevalent in most of the populations with a rising incidence over the past decade across most of the countries. An analysis of 43 populations revealed that the incidence of prostate cancer was the highest in the United States of America (USA) while the lowest incidence was reported in Asian countries [2]. The disease usually affects elderly populations compared to the youngest ones with the highest incidence in men after 60 years old [2].

Diagnosis of prostate cancer is essential for the prevention of long term complications especially mortality if the management was not appropriate [3]. Prostate-specific antigen (PSA) was presented for many years as a widely used laboratory parameter for the diagnosis of prostate cancer and its progression through the continuous rise of it is titer [1]. However, recent research inquired about the specificity of PSA in prostate cancer diagnosis especially with the PSA rise in certain diseases such as benign prostatic hyperplasia (BPH) [4]. Moreover, the invasive method of prostate cancer diagnosis by obtaining prostate biopsy was considered to be non-beneficial especially in asymptomatic patients and associated with several complications such as pain and hematospermia [5].

The strategy of active surveillance (AS) of prostate cancer entails a way for expectantly managing selected men with possible curative treatments in cases of disease progression [6,7]. Low-risk prostate cancer men, who are amenable to the AS, are identified using favorable preoperative parameters including clinical stage, tumor extent, prostate volume, and PSA [[8], [9], [10]]. However, all of these parameters have shown different limitations and accuracy deficiencies; including the re-classification risks, repeated biopsies complications, and the potential missing of the curability window [11]. Though, magnetic resonance imaging (MRI) technique was adopted as a non-invasive technique for prostate cancer diagnosis and for estimating it is progression [12]. MRI was found to be as effective as traditional methods and in some studies was reported to be superior to PSA and biopsy techniques [13,14]. In this systematic review, we aimed to review current literature about the usage of MRI examination in men with low-risk prostate cancer during active surveillance.

## 2 Methods

### 2.1 Search strategy and study selection

We performed this systematic review and meta-analysis according to the Preferred Reporting Items for Systematic Review and Meta-analyses statement (PRISMA) recommendations [15]. After collecting the appropriate keywords for developing a search term “(prostate cancer) AND (active surveillance) AND (MRI OR magnetic resonance imaging)”, we performed the systematic search for collecting relevant studies. We also performed a manual search for missed papers using the methods of Vasser and colleagues [16].

The search term was used through seven databases reported as the following: Pubmed, Google Scholar, Scopus, Web of Science, The New York Academy of Medicine (NYAM), Virtual health library (VHL), and the System for Information on Grey Literature in Europe.

Studies should be to meet the following inclusion criteria [1]: original studies [2]; assessing the value of MRI in the AS of low-risk prostate cancer [3]; patients older than 18 years [4]; the target assessment outcomes included the performance of multiparametric MRI (mpMRI) in the prediction of the disease progression (upstaging, upgrading, and unfavorable disease), which is the main outcome, the prediction ability of MRI when combined with biopsy (MR-guided biopsy), and how unnecessary MR-guided biopsies should be reduced. [5]; published in the last 5 years. We did not imply restrictions to study design, the language of the included papers, and the race of the included patients. The exclusion criteria were [1]: no report of the desired outcomes [2], intermediate and high risk of prostate cancer [3], published before 2016 [4], animal and in vitro studies and duplicate studies”.

The rationale for this 5-year limitation is mainly to give an updated piece of literature (used in many studies before [[17], [18], [19], [20]]), avoid the changing incidence and prevalence rates over years (which would affect screening results) [[21], [22], [23]], and the effect of rapidly developing MRI techniques, sequences and prostate imaging reporting/data system updates [24,25]. Moreover, the availability and access to diagnostic and health-care services as well as recommendations regarding prostate cancer screening are changing over the years [23].

The steps of title and abstract screening and full-text screening were done by five reviewers. A senior author was responsible for solving the conflicts between the five reviewers.

### 2.2 Data extraction

Three authors made a pilot extraction of few included studies for constructing a data extraction sheet. Then, another five reviewers retrieved the needed data from each of the included papers. A senior author was responsible for solving conflicts between the three extractors.

### 2.3 Risk of bias

The Institutes of Health (NIH) quality assessment tool is a widely used tool for measuring the quality of evidence [26]. Based on the included studies, we have used the tool of cross-sectional and the cohort studies reported in the NIH. The disagreement was solved through discussion between the five reviewers.

## 3 Results

### 3.1 Search results

**Fig. 1:**
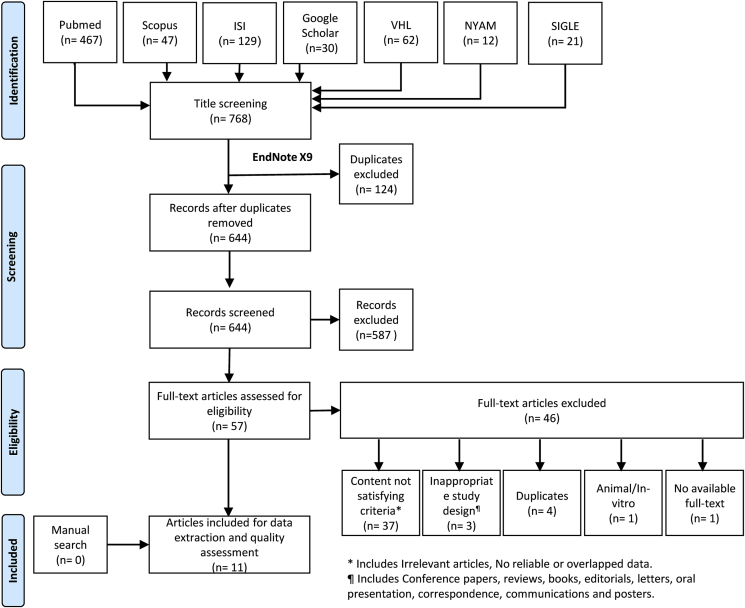
PRISMA flowchart of study search and selection process.

768 reports resulted from the database search. 644 were screened using the title and abstract screening method followed by the screening of 57 full texts for retrieving the relevant papers. We found 11 studies (Fig. 1**)**. No studies were found after performing a manual search.

### 3.2 Study characteristics and quality of the included studies

The total sample size was 1237. There were 6 prospective cohorts and 5 retrospective cohorts. Age was reported in all studies; while only one study did not report the criteria for the diagnosis of low-risk prostate cancer. All included studies had fair quality with reporting most of the main items (Table 1).

**Table 1:** Characteristics of the included studies.

Reference ID	Study design	Sample size	Mean age (SD)	Definition of low-risk prostate cancer	Quality assessment
Chen/2018/Singapore [27]	Prospective cohort	19	65.4 (4.9)	prostate specific antigen (PSA) ≤10 ng/mL, Gleason score ≤6, clinical stage ≤ T2a)	Fair
Alberts/2017/Netherlands [28]	Prospective cohort	210	65.4#	Gleason score 3 + 3	Fair
Almeida/2016/Italy [29]	Prospective cohort	73	63 (5.9)	1) men should have a histologically proven adenocarcinoma of the prostate, and they should be fit for possible curative treatment, be willing to attend the follow-up visits, and should not have received former therapy [2]; clinical stage T1c/T2 [3]; GS ≤ 6 [4]; ≤ 2 positive biopsy cores [5]; PSA ≤ 10 ng/mL [6]; PSAD ≤ 0.2 ng/mL/ml.	Fair
Borkowetz/2017/Germany [30]	Retrospective cohort	83	73#	≤cT2c, ≤2 cores with proven cancer, Gleason score ≤6 (3 + 3), prostate specific antigen (PSA) density <0.2 ng/mL2 and PSA <10 ng/mL.	Fair
Hamoen/2018/Netherlands [31]	Prospective cohort	111	64#	PSA density <0.2 ng/mL/ml, clinical stage cT1c–cT2c, and GS 3 + 3 and 2 positive biopsy cores at initial TRUSGB were	Fair
Hashimoto/2012/Japan [32]	Retrospective cohort	11	65#	NR	Fair
Hsiang/2019/USA [12]	Retrospective cohort	122	63#	grade group [GG] 1	Fair
Osses/2020/Netherlands [33]	Retrospective cohort	111	66#	ISUP grade 1	Fair
Ploussard/2019/France [11]	Retrospective cohort	143	64.4	GG 1, T1–T2 disease and PSA ≤ 10	Fair
Schoots/2018/Netherlands [34]	Prospective cohort	331	67#	(GS 3 + 3)	Fair
Vos/2016/Canada [13]	Prospective cohort	23	65	Gleason score ≤ 6, and either clinical stage ≤ T2a or PSA ≤ 10 ng/mL.	Fair

# = median, NR = not reported.

## 4 Role of multiparametric magnetic resonance imaging (mpMRI) in predicting disease progression

### 4.1 Prediction of disease upgrading

In a study by Hsiang et al., 44.3% of men who performed serial mpMRI examinations showed a progression in the subsequent imaging [12]. The parameters of performance in detecting the disease upgrade were: 41.3% sensitivity, 54.8% specificity, 22.2 positive predictive value (PPV), and 75% negative predictive value (NPV) [12]. In Almeida et al., the mpMRI showed a reasonable performance of sensitivity (76%) in detecting disease upgrading; however, there was no statistically significant correlation between clinical/pathological features and disease upgrading [29] (Table 2).

**Table 2:** Performance of multiparametric magnetic resonance imaging (mpMRI) progression by different criteria and clinical data for prediction of disease upgrading, compared to the final pathology data.

		% Sensitivity (95% CI)	% Specificity (95% CI)	% PPV (95% CI)	% NPV (95% CI)	% Accuracy (95% CI)	Odds ratio (95% CI)	AUC (95% CI)
PSA density ≥0.15 at the follow-up biopsy (12)		44.8 (26.4–64.3)	72 (61.7–80.8)	33.3 (22.9–45.6)	80.7 (74.6–85.6)	65.5 (56.4–73.9)	2 (0.8–4.9)	0.58 (0.46–0.70)
mpMRI any progression (12)		41.3 (23.5–61)	54.8 (44.1–65.1)	22.2 (14.9–31.7)	75 (67.7–81)	51.6 (42.4–60.7)	0.8 (0.3–1.9)	0.51 (0.39–0.63)
mpMRI lesion number progression (12)		17.2 (5.8–35.7)	79.5 (69.9–87.2)	20.8 (9.7–39.1)	75.5 (71.7–78.9)	64.7 (55.5–73.1)	0.8 (0.2–2.4)	0.51 (0.39–0.63)
PI-RADS score	PI-RADS score progression (12)	31 (15.2–50.8)	77.4 (67.5–85.4)	30 (18.1–45.3)	78.2 (73.3–82.4)	66.3 (57.2–74.6)	1.5 (0.6–3.8)	0.54 (0.41–0.66)
	(2–3 vs. 4–5) (29)	76	43	52	68	-	-	-
mpMRI index doubling (12)		20.6 (7.9–39.7)	68.8 (58.3–78)	17.1 (8.7–30.9)	73.5 (68.8–77.8)	57.3 (48.1–66.2)	0.5 (0.2–1.5)	0.55 (0.43–0.66)
Clinical stage (29)		15^a^	80	38	53	-	-	-
Imaging-based index of suspicion (Score 1-5) (13)		58.3	81.8	-	-	-	-	-
Clinical Grade (33)	ISUP grade ≥2	85.71 (69.74-95.19)	56.58 (44.71-67.92)	47.62 (40.48-54.85)	89.58 (78.86-95.20)	65.77 (56.16-74.51)	-	-
	ISUP grade ≥2 + cribriform growth/intraductal carcinoma Pca	82.35 (56.57-96.20)	47.87 (37.46-58.43)	22.22 (17.57-27.70)	93.75 (84.02-97.72)	53.15 (43.45-62.69)	-	-
	ISUP grade ≥3 PCa	80.00 (28.36-99.49)	44.34 (34.69-54.31)	6.35 (4.06-9.79)	97.92 (88.94-99.64)	45.95 (36.45-55.67)	-	-
Positive core (29)		36^a^	53	39	50	-	-	-
BMI, kg/m2 (29)	Cut-off 25	85^a^	28	49	69	-	-	-
	Cut-off 30	27^a^	85	60	59	-	-	-

AUC = area under the curve; CI = confidence interval; mpMRI = multiparametric magnetic resonance imaging; BMI = body mass index; NPV = negative predictive value; PIRADS = Prostate Imaging Reporting and Data System; PPV = positive predictive value; PSA = prostate-specific antigen.

a Sensitivity (as reported in the study); ISUP =International Society of Urological Pathology.

In Vos et al., the detection of prostate cancer at baseline, through MRI imaging, was not adequate, with only 43.5% sensitivity [13]. On the other hand, the prediction of disease upgrading showed better performance with a sensitivity of 58.3% and specificity of 81.8% [13]. The performance of mpMRI progression by different criteria, to predict disease upgrading, is presented in Table 2.

Schoots et al. found that 25% of men on MRI-AS showed upgrading from Gleason score (GS) 3 + 3; out of them, 71% upgraded to GS 3 + 4, 16% to GS 4 + 3, and 13 to GS ≥ 4 + 4 [34]. Additionally, in patients with a suspicious MRI index lesion, 41% of them showed upgrading from GS 3 + 3 to GS 3 + 4 or higher, 22% of Prostate Imaging Reporting and Data System (PIRADS)-3 lesions upgraded to GS 3 + 4, and 8% of PIRADS-3 upgraded to GS 4 + 3 [34].

Noteworthy, the mpMRI ability to detect the upgrading in AS of prostate cancer patients remained stable in patients with testosterone replacement therapy, without biopsy progression [32].

### 4.2 Prediction of disease upstaging

The mpMRI showed an appropriate sensitivity (92%) to detect disease upstaging, with a higher NPV compared to upgrading (96% Vs. 68%) [29]. Moreover, disease upstaging was significantly correlated to patients’ age, clinical stage, and visible disease [29]. Vos et al., also found an adequate sensitivity (100%) of mpMRI to detect disease upstaging, however, the specificity was lower, down to 30% [13] (Table 3).

**Table 3:** Performance of multiparametric magnetic resonance imaging (mpMRI) progression by different criteria and clinical data for prediction of disease upstaging, compared to the final pathology data.

		% Sensitivity	% Specificity	% PPV	% NPV
Clinical stage [29]		38	87	38	87
Imaging-based index of suspicion (Score 1–5)	**1**–**5** [13]	100	30	–	–
	**1**–**2** [31]	–	–	–	85
	**≥ 3** [31]	71^a^	–	–	–
Positive core [29]		54	60	23	86
PIRADS (2–3 vs. 4–5) [29]		92	40	25	96
BMI, kg/m2 [29]	**Cut-off 25**	85	23	23	88
	**Cut-off 30**	38	83	33	86

BMI: body mass index; NPV = negative predictive value; PIRADS = Prostate Imaging Reporting and Data System; PPV = positive predictive value.

a Sensitivity.

In the same context, Hamoen et al. adopted an imaging-based index of suspicion (Score 1 to 5) to evaluate the MRI role [31]. They found that patients with score ≤2 had an NPV of 85% for detecting disease upstaging, compared to a sensitivity of 71% in patients with scores ≥3 [31] (Table 3). The same study highlighted that mp-MRI and MR-guided biopsy (MRGB) had a good predictive value limited to the first year; however, transrectal ultrasound-guided biopsy (TRUSGB) showed a superior role in detecting patients with a GS ≥ 7, following the first year [31].

### 4.3 Prediction of unfavorable disease

The unfavorable disease was defined as the presence of upgrading and/or upstaging, and PIRADS score >3. The mpMRI showed an intermediate sensitivity (76%) to detect unfavorable disease, with a specificity of 44% and PPV of 58%. The unfavorable disease had a lower NPV compared to upstaging and upgrading (64% Vs. 96% Vs. 68%) [29]. Additionally, the unfavorable disease was significantly correlated to PIRADS-5 [29] (Table 4).

**Table 4:** Performance of multiparametric magnetic resonance imaging (mpMRI) progression by different criteria and clinical data for prediction of unfavorable disease, compared to the final pathology data [29].

		% Sensitivity	% Specificity	% PPV	% NPV
Clinical stage		16	81	46	48
Positive core		38	53	45	45
PIRADS (2–3 vs. 4–5)		76	44	58	64
BMI, kg/m2	**Cut-off 25**	84	28	54	63
	**Cut-off 30**	27	86	67	53

BMI: body mass index; NPV = negative predictive value; PIRADS = Prostate Imaging Reporting and Data System; PPV = positive predictive value.

### 4.4 Role of MR-guided biopsy (MRGB) in predicting disease progression

A combination of mp-MRI and MRGB would is of additional value in the AS process of prostate cancer patients, especially during the first year [31]. This combination re-classified 23% of the patients, with 60% of the re-classified due to GS increase [31]. In the same context, with PSA-density (PSA-D) cut-off 0.15 ng/mL^2^, all PIRADS-3 lesion with upgrades to GS ≥ 3 + 4 were detected in patients with PSA-D ≥0.15 ng/mL^2^ [34]. The number of positive MRIs with GS outcome of MRGB stratified to PI-RADS and PSA-D is summarized in Table 5.

**Table 5:** Number of positive MRIs with Gleason score outcome of MRI-targeted biopsies, stratified to PI-RADS and PSA-density.

	Schoots/2018/Netherlands [34]		Alberts/2017/Netherlands [28]	
	PSA Density (N = 198)		PSA Density (N = 210)	
	<0.15	≥0.15	<0.15	≥0.15
PI-RADS				
3	36%	64%	44%	56%
4	43%	57%	37%	63%
5	28%	72%	21%	79%
Gleason score (GS)				
No PCa	62%	38%	49%	51%
GS 3 + 3	46%	54%	–	–
GS 3 + 4	22%	78%	19%	81%
GS 4 + 3	8%	92%	–	–
GS ≥ 4 + 4	18%	82%	–	–
GS ≥ 3 + 4	20%	80%	–	–
GS ≥ 4 + 3	12%	88%	7%	93%

PIRADS = Prostate Imaging Reporting and Data System; PSA-D = prostate specific antigen-density; PCa = prostate cancer.

In terms of biopsy scheme used to assess prostate cancer upstaging and upgrading, targeted biopsies alone would miss 21.7% of cancer lesion; out of them, 16.7% are of grade group (GG) ≥ 3 [11]. However, a combination of targeted and systematic biopsies would lower the risk of GG ≥ 3 disease by 39%, compared with targeted biopsies alone [11]. Noteworthy, the biopsy scheme did not have a significant effect on the upstaging rates, even with the combination of targeted and systematic biopsies [11]. Borkowetz et al. reported similar results for combination biopsies, where a combination of MRI/ultrasound-fusion biopsy and systematic biopsy, in patients undergoing AS for prostate cancer, outperformed both modalities alone [30]. The combination scheme detected upgrading in 71% if the patients compared to 64% and 59% of MRI/ultrasound-fusion and systematic biopsies, respectively [30]. Another suggested combination scheme is the MRI-targeted and transperineal template biopsies, which detected disease upgrading in 26.3% of the patients, outperforming any of the two types alone [27].

### 4.5 Strategies to reduce unnecessary MRGB

In 59% of men with suspicious MRI lesions, MRGB did not show upgrading. Those biopsies could be considered unnecessary and harmful, especially in patients with PIRADS-3 (70% GS 3 + 3 or no prostate cancer) [34]. Similarly, 64% of PIRADS-4 and 34 of PIRADS-5 MRGBs were found to be unnecessary [34]. For that, Schoots et al. have suggested some possible strategies to reduce this possible harm as seen in Table 6.

**Table 6:** Possible strategies to reduce targeting biopsies in low-risk men in active surveillance [34].

	**No targeted biopsy**	**Targeted biopsy**	**No targeted biopsy**	**Targeted biopsy**
MRI index lesions	Threshold csPCa: GS ≥ 3 + 4		Threshold csPCa: GS ≥ 4 + 3	
Stratification into PSAD <0.15 and ≥ 0.15 ng/mL^2^				
PI-RADS 3	P3 and PSA-D <0.15	P3 and PSAD ≥0.15	P3 and PSA-D <0.15	P3 and PSAD ≥0.15
PI-RADS 4		P4 and any PSAD	P4 and PSA-D <0.15	P4 and PSAD ≥0.15
PI-RADS 5		P5 and any PSAD		P5 and any PSAD
Stratification into PSAD <0.20 and ≥ 0.20 ng/mL^2^				
PI-RADS 3	P3 and PSA-D <0.20	P3 and PSAD ≥0.20	P3 and PSA-D <0.20	P3 and PSAD ≥0.20
PI-RADS 4		P4 and any PSAD		P4 and any PSAD
PI-RADS 5		P5 and any PSAD		P5 and any PSAD

PIRADS = Prostate Imaging Reporting and Data System; PSA-D = prostate specific antigen-density; csPCa, clinically significant prostate cancer; GS, Gleason score.

## 5 Discussion

The trials to use MRI to identify tumor locations in prostate cancer have started as early as the 1980s, using T1-weighted and T2-weighted images which lacked sensitivity and specificity [35]. The role of mpMRI was traditionally confined to prostate cancer staging and was typically done following biopsy to assess the possibility of different treatment modalities [36]. Recently, the function of mpMRI expanded to include tumor identification, monitoring disease during AS, and follow-up of the patients [36].

In the current study, we are presenting different aspects of mpMRI and MRGB performance as a part of the AS process. Our results showed an adequate sensitivity and specificity of both modalities to detect disease progression; including disease upgrading and upstaging. Moreover, the mpMRI ability to detect the progression in AS of prostate cancer patients remained stable in patients with testosterone replacement therapy, without biopsy progression. However, the performance in the prediction of unfavorable disease was inferior to the detection of upgrading and upstaging. In terms of MRGB, the previous literature agreed on the superiority of using a combination of different biopsy schemes to get a better progression section. Noteworthy, mp-MRI and MRGB had a good predictive value limited to the first year, with TRUSGB showing a superior role in detecting patients with a GS ≥ 7, after that.

Prostate cancer traditional identification is done using TRUSGB; nevertheless, it showed a low detection rate of 27%–44%, over-diagnosis of non-significant lesions, and missing some important ones, especially in the anterior portion of the prostate [[37], [38], [39]]. In addition to the aforementioned advantages in mpMRI performance, it can be used to target the identified lesion, either by MRGB or MRI/ultrasound-fusion biopsies [40,41]. Both of MRGB and MRI/ultrasound-fusion biopsies have higher accuracy when compared to TRUSGB alone [[42], [43], [44], [45]]. A previous systematic review showed that MRGB is superior to TRUSGB with a third fewer biopsy indicated and a 10% fewer detection of clinically insignificant lesions [46]. This was also confirmed by other studies that found a reduced missing of the clinically significant lesions using MRGB compared to TRUSGB [47], with a tumor detection rate of 70.1% in MRGB, compared to only 13.1% for TRUSGB [48].

In terms of assessing disease aggressiveness and staging, mpMRI showed a higher performance and accuracy in staging localized prostate cancer, when compared to the Partin table [49]. In the same context, MRI T2w imaging and dynamic contrast enhanced-MRI have shown high accuracy in staging prostate cancer and identifying tumors extending beyond prostate boundaries (T3 stage) [50,51]. Using mpMRI can also help in choosing treatment strategy in patients with low-risk prostate cancer to help with planning radiotherapy and surgery [52]. Moreover, mpMRI can be used to assess tumor volume, extension, and location, which is useful information to guide focal therapy [53]. Although some evidence is present on how mpMRI may miss some secondary satellite lesions, further examination of these lesions concluded that they were low-grade and significantly small ones [54].

The current study has some limitations affecting the generalizability of conclusions. A few studies did not provide a detailed definition of low-risk prostate cancer and there is some relevant heterogeneity among those who did. Although all studies concluded the usefulness and added value of MRI in AS, the performance of mpMRI and MRGB is variable among different studies.

## 6 Conclusion

Both of mpMRI and MRGB have shown an adequate performance on assessing disease progression in the AS of low-risk prostate cancer patients. They can be used for disease staging and grading for successful treatment planning.

## Sources of funding

None.

## Ethical approval

As per hospital Ethical Committee protocol, Systematic reviews and Meta analyses do not need approval as they are considered pre-approved.

## Consent

Non applicable.

## Author contribution

All authors were part of the study design, data collection, data analysis, interpretation, writing, editing, language proofing and resource checking of the paper.

## Registration of Research Studies

Name of the registry: Research Registry.

Unique Identifying number or registration ID: reviewregistry921.

Hyperlink to your specific registration (must be publicly accessible and will be checked): https://www.researchregistry.com/register-now#user-systematicreviewmeta-analysesregistry/registerasystematicreviewmeta-analysidetails/5ed7b051f7f53c0015528bf8/.

## Guarantor

Sultan Zaher Alshehri.

Omar Safar Alshahrani.

## Provenance and peer review

Not commissioned, externally peer reviewed.

## Please state any conflicts of interest

The authors declare no conflict of interest.

## Please state any sources of funding for your research

No funding was granted.

## Ethical approval

As per hospital Ethical Committee protocol, Systematic reviews and Meta analyses do not need approval as they are considered pre-approved.

## Consent

Non applicable.

## Author contribution

All authors were part of the study design, data collection, data analysis, interpretation, writing, editing, language proofing and resource checking of the paper.

## Registration of research studies

1.Name of the registry: Research Registry.

2.Unique Identifying number or registration ID: reviewregistry921.

3.Hyperlink to your specific registration (must be publicly accessible and will be checked): https://www.researchregistry.com/register-now#user-systematicreviewmeta-analysesregistry/registerasystematicreviewmeta-analysidetails/5ed7b051f7f53c0015528bf8/

## Guarantor

Sultan Zaher Alshehri.

Omar Safar Alshahrani.

## Declaration of competing interest

On behalf of all authors, the corresponding author states that there is no conflict of interest.
